# Ferritin measurement IN Donors—Effectiveness of iron Monitoring to diminish iron deficiency and low haemoglobin in whole blood donors (FIND’EM): study protocol for a stepped wedge cluster randomised trial

**DOI:** 10.1186/s13063-020-04648-w

**Published:** 2020-10-01

**Authors:** Maike G. Sweegers, Saurabh Zalpuri, Franke A. Quee, Elisabeth M. J. Huis in ‘t Veld, Femmeke J. Prinsze, Emiel O. Hoogendijk, Jos W. R. Twisk, Anton W. M. van Weert, Wim L. A. M. de Kort, Katja van den Hurk

**Affiliations:** 1 Donor Studies, Sanquin Research, Amsterdam, The Netherlands; 2 Department of Cognitive Science & Artificial Intelligence, Tilburg University, Tilburg, The Netherlands; 3 Department of Epidemiology and Biostatistics, Amsterdam Public Health Research Institute, Amsterdam UMC – Location VU University Medical Centre, Amsterdam, The Netherlands; 4 National Screening Laboratory Sanquin, Sanquin Research and Labservices, Amsterdam, The Netherlands

**Keywords:** Blood donation, Donation interval, Donor health, Stepped wedge cluster randomised trial, Ferritin

## Abstract

**Background:**

Blood donors are at risk for reduced iron stores, because of which donor iron monitoring received increased attention in the last decade. Despite the importance for donor health, international consensus on an appropriate policy for iron monitoring is lacking. Therefore, we conduct a trial to evaluate to what extent ferritin-guided donation intervals are effective in increasing haemoglobin and ferritin levels, decreasing low-haemoglobin deferral, increasing donor return and improving the health of whole blood donors in the Netherlands.

**Methods:**

Sanquin Blood Bank is implementing ferritin-guided donation intervals to prevent donors from increasing iron loss at repeated donations. Using a stepped wedge cluster randomised trial approach, the design involves a random crossover of 29 clusters of blood collection centres from the existing policy without ferritin measurements to a ferritin-guided donation interval policy. This new policy includes ferritin measurements for all new donors and at every 5th whole blood donation, extending donation intervals to 6 months if ferritin is 15–≤ 30 ng/mL and to 12 months if ferritin is < 15 ng/mL. We measure ferritin levels of whole blood donors from stored plasma samples and collect haemoglobin levels and information on low-haemoglobin deferral and donor return from the donor database before, during and after the implementation period. We measure donor health during and after the implementation period using questionnaires, assessing physical and mental wellbeing and iron deficiency- and donation-related symptoms. We use multilevel analyses to investigate differences in ferritin and haemoglobin levels, low-haemoglobin deferral rates, donor return and donor health from whole blood donors, between blood collection centres that have versus those that have not yet implemented the ferritin-guided donation interval policy.

**Discussion:**

This stepped wedge cluster randomised trial will provide insight into the effectiveness of ferritin-guided donation intervals in lowering iron deficiency, decreasing donor deferrals due to low haemoglobin and improving donor health. We will evaluate a policy that is implemented nationwide in a real-life setting. Our study is therefore not limited to a small experimental setting and the results will guide policymakers seeking an appropriate policy for iron monitoring.

**Trial registration:**

The Dutch trial registry NTR6738. Registered on 29 September 2017. Retrospectively registered.

**Table:** 

**Title**	Ferritin measurement IN Donors – Effectiveness of iron Monitoring to diminish iron deficiency and low haemoglobin in whole blood donors (FIND’EM): study protocol for a stepped wedge cluster randomised trial
**Trial registration**	https://www.trialregister.nl/trial/6549
**Protocol version**	**3**
**Funding**	‘Product and Process Development Cellular Products Grant’ (PPOC18-15) granted to K. van den Hurk
**Author details**	1. Donor Studies, Sanquin Research, Amsterdam, the Netherlands; 2. Department of Cognitive Science & Artificial Intelligence, Tilburg University, Tilburg, The Netherlands 3. Department of Epidemiology and Biostatistics, Amsterdam Public Health research institute, Amsterdam UMC – Location VU University medical centre, Amsterdam, the Netherlands 4. National Screening Laboratory Sanquin, Sanquin Research and Labservices, Amsterdam, the Netherlands;
**Name and contact information for the trial sponsor**	Research Programming Committee, Sanquin, Amsterdam, The Netherlands Plesmanlaan 125 NL-1066 CX Amsterdam The Netherlands Telephone +31 20 512 30 00 Fax +31 20 512 33 03
**Role of sponsor**	The sponsor has no role in study design, writing of the report and decision to submit the report for publication.

## Introduction

As a result of one donation, whole blood donors lose 8% (men) to 81% (menstruating women) of their total iron stores [1–3]. High donation frequency increases the risk for depleted iron stores through the haemoglobin (Hb)-bound iron loss and subsequent increased erythropoiesis [4, 5]. Depletion of iron stores, iron-deficient erythropoiesis and iron-deficiency anaemia may not only negatively influence donor health [6], but may also lead to a higher risk of low-Hb deferral [7], which is demotivating for a blood donor and costly for blood banks [8]. Most blood banks monitor donors’ Hb levels and defer donors with Hb levels below a certain threshold, aiming to avoid anaemia, minimise low-Hb deferral and ensure sufficient Hb content for transfusion [9]. However, these Hb levels do not reflect the donors’ iron status [5, 10] and the World Health Organization advises the monitoring of serum ferritin to detect iron deficiency [11].

Iron deficiency in whole blood donors can be prevented by extending donation intervals or providing iron supplements [5, 9, 12]. Extending donation intervals might be the preferred method in light of possible gastrointestinal disturbances as a result of iron supplementation, poor compliance or negative perceptions of taking iron tablets [12]. Previous studies have shown that prolonged donation intervals are associated with lower risk of low-Hb deferrals [8, 13]. Additionally, the results from the randomised-controlled INTERVAL trial have shown that donating at 8-week intervals for men and 12-week intervals for women lead to significantly lower Hb and ferritin concentrations than those observed in longer donation interval groups (i.e. 12-week intervals and 16-week intervals for men and women, respectively) [8].

Sanquin Blood Bank, solely responsible for the collection and distribution of blood products in The Netherlands, decided to implement a new policy for whole blood donors based on ferritin-guided donation intervals. This policy includes the measurement of ferritin levels, in addition to the regular Hb measurement, prior to the first donation of each new donor and after every fifth whole blood donation. If ferritin levels are low, donation intervals are extended to 6 or 12 months. In order to enable a scientific and methodologically sound evaluation of its effectiveness, limit the anticipated (temporary) impact on donor availability and to provide logistical benefits to the implementation of the new policy, Sanquin Research advised the Blood Bank to implement the ferritin-guided donation interval policy following a stepped wedge approach, involving random cross-over of blood collection centres from the existing policy without ferritin measurement to the policy including ferritin-guided donation intervals.

### Research objectives

The FIND’EM study evaluates, by means of a nationwide stepped wedge cluster randomised trial, the effects of the ferritin-guided donation interval policy for whole blood donors on (1) Hb and ferritin levels, (2) low-Hb deferral, (3) donor return rates and (4) donor health (i.e. physical and mental wellbeing and iron deficiency- and donation-related symptoms) [14]. We anticipate that the implementation of routine ferritin measurement in whole blood donors and extension of the donation interval in case of low ferritin levels will result in a decrease in low-Hb deferral rates and increase in ferritin and Hb levels, donor health and return rates.

## Method

### Setting

The Sanquin Blood Bank is the only organisation authorised to collect, prepare and distribute blood products, in the Netherlands. All donations are voluntary and non-remunerated. Sanquin consists of 50 fixed blood collection centres, organised into blood collection clusters, 29 in total, distributed over four different topographic regions. Each cluster consists of one to three fixed blood collection centres, some also including mobile collection centres. New donors have to undergo an eligibility test before their first blood donation. This pre-donation screening includes checking of health conditions and risk behaviours by means of a donor health questionnaire and interview, blood pressure and Hb testing and blood sampling for blood typing and infectious disease testing. When eligible, men can subsequently donate a maximum of 5 times/year (minimum donation interval of 56 days) and women a maximum of 3 times/year (minimum donation interval of 120 days).

### Study design

**Fig. 1:**
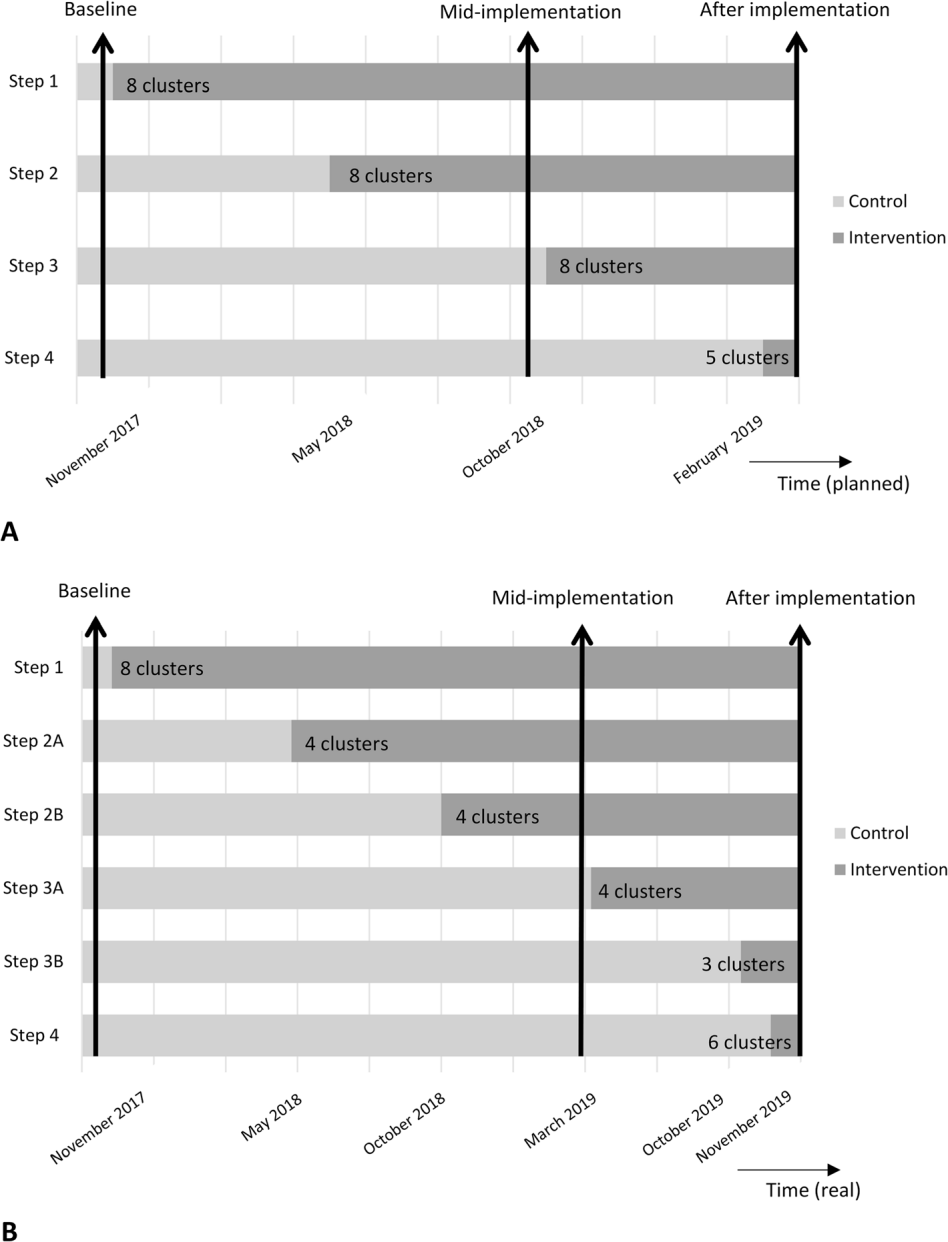
Study design of FIND’EM, a stepped wedge cluster randomised trial. Bars indicate the various time points at which the indicated number of blood collection clusters shifted from current practice (control) to ferritin-guided donation intervals (intervention) during the implementation period of the policy. The vertical arrows indicate the three measurement time points. **a** Planned timing of implementation. **b** Real timing of implementation

The FIND’EM study is a stepped wedge cluster randomised trial involving whole blood donors from all 50 fixed blood collection centres in the Netherlands. The stepped wedge cluster randomised design is increasingly being used in the evaluation of ‘service delivery type interventions’, which are bounded by logistical or policy constraints. A stepped wedge design is a one-way crossover trial in which several allocation groups start at different time points with the implementation of the new policy [14]. For FIND’EM, this involves random and sequential crossover of clusters from the current policy without ferritin measurements to the policy including ferritin-guided donation intervals, until all clusters are exposed to this new policy (Fig. 1). The numbers 1 to 29 have been randomly assigned to one of the 29 blood collection clusters. Allowing only a maximum of two clusters of a similar topographic region to each implementation step, the two clusters with the lowest numbers from each region were allocated to step 1, the clusters with the third and fourth lowest numbers from each region were allocated to step 2, the clusters with the fifth and sixth lowest numbers from each region were allocated to step 3 and the remaining clusters were allocated to step 4. The allocation was not concealed and the planned timing of crossover of all blood collection clusters was revealed before the start of the implementation period. In November 2017, Sanquin started with implementing ferritin-guided donation intervals in eight clusters of blood collection centres (28% of all blood collection centres). The next three implementation steps were planned for May 2018, October 2018 and February 2019. The number of repeat whole blood donors with low ferritin levels appeared to be higher than anticipated. Therefore, to ensure sufficient blood supply in The Netherlands, further implementation had to be delayed and executed in smaller steps. The second step of implementation thus included four clusters of collection centres from different topographic regions (step 2a), instead of the planned eight clusters, and was scheduled for May 2018. The remaining clusters of step two implemented ferritin-guided donation intervals in October 2018 (step 2b). The third step was again split into two implementation waves, in which four clusters implemented the policy in March 2019 (step 3a) and three additional clusters of step 3 started in October 2019 (step 3b). Due to logistical reasons, one cluster randomised to step 3 was not able to implement the ferritin-guided donation intervals in October 2019. The fourth and final step included the remaining six clusters of collection centres and was executed in November 2019 (Fig. 1). On average, a total of 1275 whole blood donations are done at collection centres of each implementation step per week. Effectively, this stepwise strategy results in a gradual increase of the intervention group and a simultaneously decreasing control group. This implementation strategy and the ferritin-guided donation interval policy itself was approved by Sanquin’s Board of Directors after review and approval by Sanquin’s Medical and Ethical Advisory Boards. Additionally, Sanquin’s Ethical Advisory Board has approved the FIND’EM protocol for additional questionnaires and ferritin measurements. As part of standard practice, blood donors are asked for consent for their unusable remainder of the donation to be used for medical scientific research. Consent for the use of this material was included in the ethical approval for the current study. All research activities commenced after ethical approval was obtained. All participating donors who filled in a questionnaire provided additional written informed consent. As the FIND’EM study is a minimal risk study, a data monitoring committee is not needed.

### Study population

**Fig. 2:**
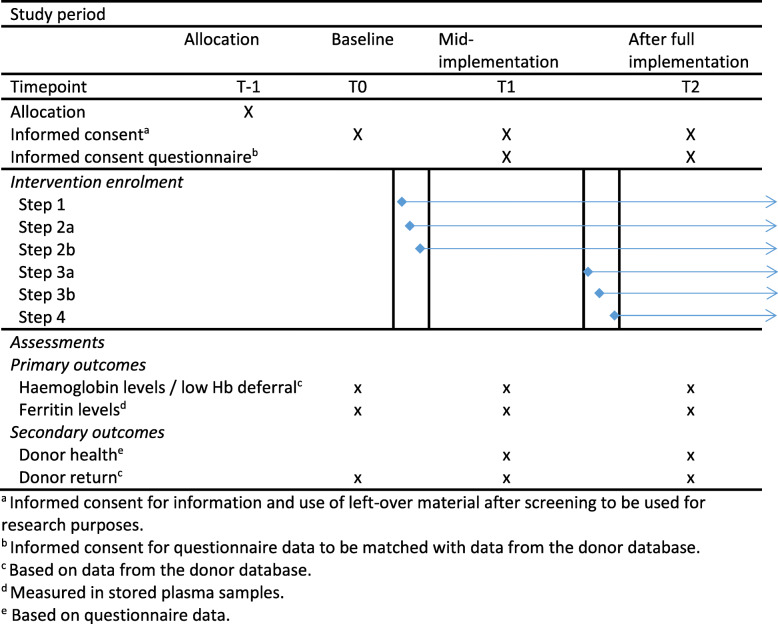
Spirit figure, schedule of intervention enrolment and assessments

Both new (i.e. a donor undergoing pre-donation screening) and repeat donors (i.e. a donor making a whole blood donation or attempt) are eligible to participate in this study. The intervention likely results in delayed effects. Ferritin levels of a donor visiting a blood collection centre where the intervention has been implemented will only be measured every fifth donation. Furthermore, the extension of donation intervals makes it necessary to allow the blood collection centres to be exposed to the intervention for a certain amount of time before donors return and the effectiveness of the new policy becomes observable. We therefore selected three measurement time points: (1) before implementation (i.e. baseline), (2) mid-implementation (when 55% of blood collection centres had implemented the policy) and (3) after complete implementation of ferritin-guided donation intervals. As Sanquin had already started the implementation of the ferritin-guided donation intervals in November 2017, before approval from the Ethical Advisory Board for the FIND’EM study, we were unable to collect questionnaire data from donors at baseline. However, baseline data on other outcomes (Hb and ferritin levels, low-Hb deferral and donor return) are measured after ethical approval in stored samples, and a large majority of the donors (99%) provide their consent to the routine request to ‘make the unusable remainder of my donation available for medical scientific research in general’ (Fig. 2). Whole blood donors who visit one of the 50 fixed blood collection centres during a pre-selected week at mid-implementation or after complete implementation are asked to participate by completing questionnaires. This makes the current study an open cohort design study with repeated cross-sectional measures and new samples at each measurement. Donors registering at the registration desk of one of the collection centres during one of these time points will receive a leaflet with information on why this study is performed, explaining that upon participation, the donor is asked to fill in a questionnaire and participation is voluntary, the leaflet contains contact details of the research team in case of any questions and a link to the study website with additional information and frequently asked questions. The donor is asked to read the study information while waiting for their health check. After the check, the donors will be asked whether they have read the study information. If the donor decides to participate, an informed consent form will be signed and the donor physician or nurse will sign the form as a representative of the researcher. Donors who decide not to participate are asked to write down the reason for non-participation.

### Control condition

Before each whole blood donation, a donor is seen by one of the doctors or nurses to check haemoglobin levels and blood pressure and screen for any indications a donor might not be eligible to donate whole blood at that moment. Sanquin’s current guidelines include Hb cut-offs for donor eligibility of > 13.5 g/dL for men and > 12.5 g/dL for women, which is in accordance with the international guidelines and European legislation [15]. Donors with low Hb (measured prior to donating in capillary blood with HemoCue 201, Angelholm, Sweden) are deferred from donating for 3 months.

### Intervention: ferritin-guided donation intervals

**Fig. 3:**
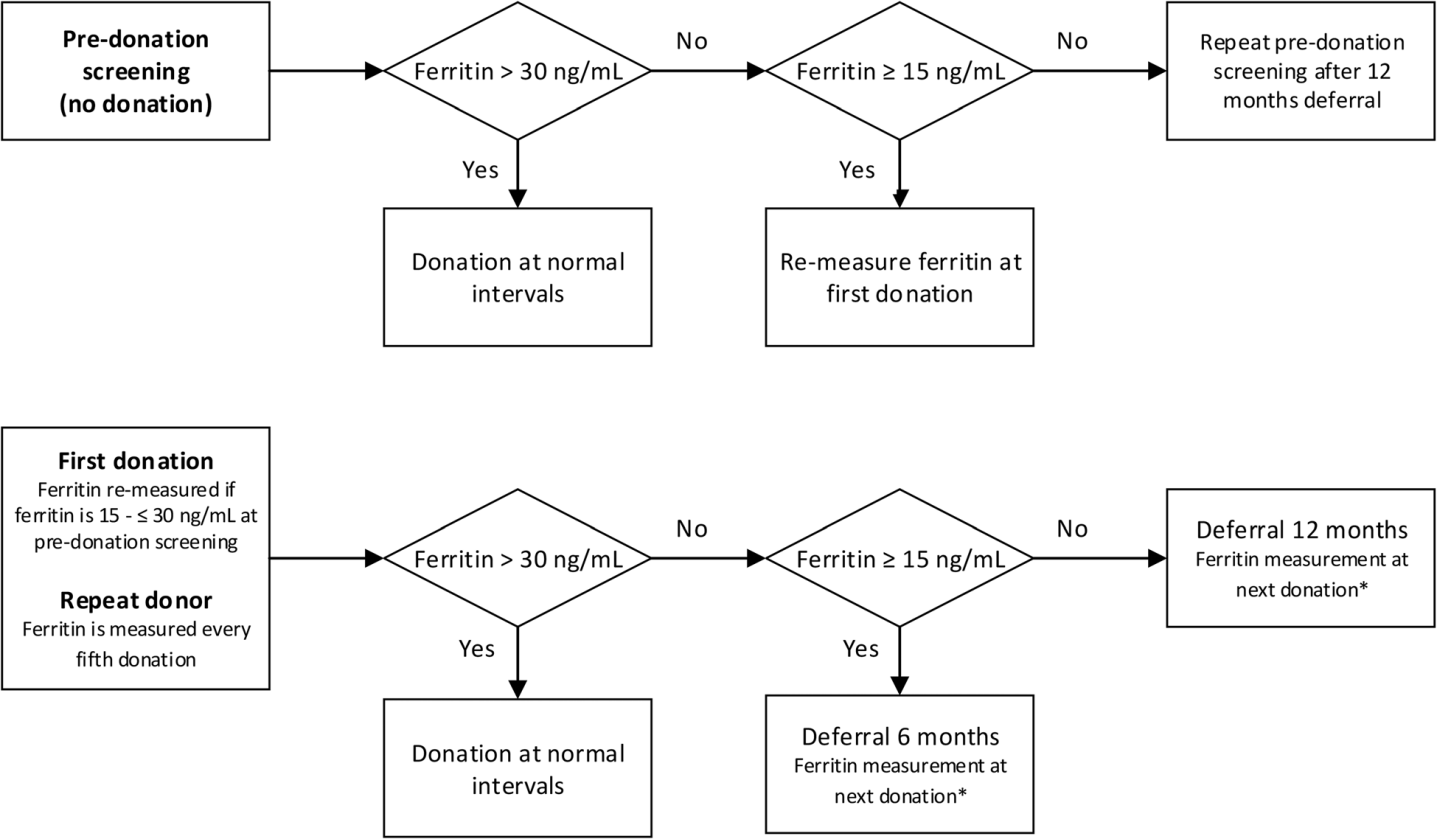
Schematic of the standard operating procedure for ferritin-guided donation intervals. Asterisk indicates that if ferritin levels were ≤ 30 ng/mL previously, ferritin levels will be measured again after 6 or 12 months deferral before the donor is allowed to donate. Deferral strategies from this ferritin screening will follow the standard operating procedure of ‘repeat donor’

The ‘intervention’ includes ferritin-guided donation intervals with ferritin measurements in whole blood donors at pre-donation screening and every 5th donation, in addition to the standard Hb measurement as described as the control condition. Ferritin level cut-offs are based on the definitions for iron deficiency (< 15 ng/mL) and reduced iron level (≤ 30 ng/mL) by Alvarez-Ossorio et al. [12]. With respect to the duration of donation intervals, it is acknowledged that iron-deficient donors may need tailored donation intervals but knowledge on optimal recovery times for iron-deficient donors is lacking. Our deferral period is based on the study by Schotten et al. in which recovery of ferritin levels in new and repeat male donors was suggested to require donation intervals of 180 days [5]. If ferritin during the pre-donation screening is higher than 30 ng/mL, the donor is allowed to donate following regular donation schedules. If ferritin is ≤ 30 ng/mL but ≥ 15 ng/mL, the donor is allowed to donate during the next visit to the blood collection centre, but ferritin will be measured again from the blood sample taken during their first whole blood donation. If ferritin during pre-donation screening is < 15 ng/mL, the donor is not allowed to donate for 12 months. After 12 months, the donor is allowed to donate but ferritin is measured again from the blood sample taken during their first whole blood donation. If the donor experiences complaints that can be associated with a low ferritin level, the new donor is advised to visit his/her general practitioner (Fig. 3).

For repeat whole blood donors, the intervention includes ferritin measurements from blood samples taken at every fifth donation. When ferritin is > 30 ng/mL, donors are eligible to donate at normal donation intervals. If ferritin is ≤ 30 ng/mL but ≥ 15 ng/mL, donors are deferred for 6 months (Fig. 3). If ferritin is < 15 ng/mL, donors are deferred from donating for 12 months (Fig. 3). After deferral, ferritin levels are re-measured from blood taken during the next donation. When ferritin is < 30 ng/mL again, the ferritin level is re-measured after 6 or 12 months deferral, before the donor is allowed to donate. The deferral policy after this re-measured ferritin level follows that of repeat whole blood donors (Fig. 3). In addition, the donor may be advised to visit his/her general practitioner and/or to donate at a lower donation frequency. This decision is made by a donor physician, based on the donor’s medical history and coinciding iron deficiency-related complaints.

### Data collection

Pseudonymized data on age, sex, Hb levels and deferrals will be extracted from the donor database (eProgesa software application; MAK-SYSTEM) for all new and repeat whole blood donors who visit a fixed blood collection centre during a pre-selected week at one of the three measurement time points and gave consent for their information and left-over material to be used for research purposes. Furthermore, we will calculate donor return rates as the percentage of donors with a donation attempt within 6 months after the next allowed donation date. We will use stored plasma samples of new donors and repeat donors who made a donation attempt at one of the fixed blood collection centres during one of the pre-selected measurement time points to determine ferritin levels in intervention and control groups. Ferritin will be measured on the Architect i2000sr (Abbott Diagnostics) from donation samples collected in K3EDTA tubes (Greiner) and stored at − 30 °C. After storage, ferritin levels may be lower than those in fresh samples [16]. Within the FIND’EM study, we will compare ferritin levels of donors donating at centres where the ferritin-guided donation interval policy was implemented to ferritin levels of donors donating at centres where the policy was not implemented yet. For both groups, we will use samples that were stored for the same time period. Therefore, we expect similar decreases of ferritin levels as a result of storage in both groups, still allowing this comparison. For the validation of ferritin levels from stored samples of donors participating in the FIND’EM study, we will compare ferritin levels from stored samples with ferritin levels measured in fresh samples as part of the ferritin-guided donation interval policy.

At mid-implementation and after full implementation, all new and repeat whole blood donors who visit fixed blood collection centres during the pre-selected week are asked to participate in additional data collection for this study and to fill in an informed consent form. Subsequently, donors are asked to complete often-used or validated questionnaires during or right after whole blood donation. These questionnaires assess physical and mental wellbeing (SF-36 questionnaire [17]), fatigue (Checklist Individual Strength) [18], donation-related symptoms [19], restless legs syndrome and pica [8], cognitive functioning (Cognitive Failure Questionnaire; supplementary file) and warm glow [20]. Questionnaires are completed online, using Qualtrics XM®, and are accessible by following a hyperlink or using the provided QR code. If donors are unable to fill in the questionnaires online, the blood collection staff provides paper questionnaires or questionnaires are sent by (e-)mail.

### Sample size calculations

Using data from the first clusters of blood collection centres that have implemented the ferritin-guided donation intervals, we found a mean increase in ferritin levels of 4 ng/mL (standard deviation (SD) 54 ng/mL) over a 6-month period. To detect a difference of 4 ng/mL, assuming a SD of 54 ng/mL, a two-sided significance level of 5%, a power of 80% and ignoring the correlated observations, a sample size of 5720 subjects is needed. We used the following formula to calculate the actual sample size:

$$ m=\frac{N}{1+\left(n-1\right)\left(1-\rho \right)} $$

where *m* = number of blood collection centres, *N* = sample size ignoring the correlated observations; *n* = average number of subjects within a blood collection centre and *ρ* = intraclass correlation coefficient. Assuming an average number of subject within each blood collection centre of 150 and an intraclass correlation coefficient of 0.1, we need 42 centres in total. With the fixed number of 50 blood collection centres (i.e. 7500 subjects), the power of the study is more than enough to detect the difference of 4 ng/mL. An individual autocorrelation coefficient is not included in our sample size calculation as we include repeated cross-sectional measures. With approximately 7500 donation attempts at fixed collection centres each week, we anticipate that we will be able to collect data on age, sex, Hb levels and deferrals and ferritin levels of approximately 7500 donors at baseline, mid-implementation and after implementation, thereby including more than sufficient data to reliably investigate the effectiveness of the ferritin-guided donation intervals. Furthermore, during data collection at mid-implementation, approximately 3500 donors filled in the questionnaires. We expect about the same response for the data collection after full implementation of the new policy.

### Data analyses

Descriptive statistics will be provided for the donor population at baseline, mid-implementation and after full implementation. Linear mixed models (i.e., multilevel analysis) will be used to investigate differences in Hb and ferritin levels, low-Hb deferral, donor return rates and donor health between intervention and control groups using data collected at baseline, mid-implementation and after implementation. Two-level models with donors nested within blood collection clusters will be fitted, with a random intercept for cluster to adjust for the correlation between measurements of donors within the same cluster. All models include a binary variable indicating whether the observation was done before or after the start of the intervention and will be adjusted for the time point of implementation of the policy (fixed variable, time in months since the start of implementation period). Because of the nature of our outcome measures and because we include a new sample of donors at every measurement time point, we do not expect robust methods to be appropriate for sensitivity analyses. To investigate how the impact of ferritin-guided donation intervals develops over time, the latter will also be included as an effect modifier [14]. An intention-to-treat approach will be used and stratified analyses will be performed to investigate differences in the effectiveness of ferritin-guided donation intervals for male and female donors.

### Trial oversight

For the implementation of ferritin-guided donation intervals, the Sanquin Blood Bank has formed a Ferritin Steering Committee including senior clinical, laboratory, managerial and academic members who monitor the overall conduct of the implementation of ferritin-guided donation intervals. The Ferritin Steering Committee provides Sanquin’s Board of Directors and Sanquin’s Medical and Ethical Advisory Boards with advice regarding the implementation, including the timing and number of clusters for each implementation step. Additionally, the Ferritin Project Committee involves staff members responsible for practical aspects of the implementation, such as communication to donors, training of blood collection staff, laboratory testing and Information Technology. Finally, the FIND’EM Research Team, consisting of researchers authoring this paper, monitors the data collection and analyses for the study. Some overlap in members of the Ferritin Steering and Project Committees and the FIND’EM Research Team enables easy information exchange and smooth coordination of activities.

## Discussion

Currently, there is no international consensus on an appropriate policy for iron monitoring in whole blood donors. To our knowledge, this is the first randomised study to evaluate the effectiveness of ferritin-guided donation intervals on Hb and ferritin levels, low-Hb deferrals, return rates and health of donors.

The use of a stepped wedge cluster randomised trial approach allows to implement and simultaneously evaluate the policy. As data collection takes place at multiple time points during the implementation period, all blood collection centres contribute data under both the control and intervention condition. Furthermore, this design makes it possible to investigate the effects as if it was a randomised controlled trial by correcting for time and clustering of donors and blood collection centres. This design also introduces some challenges, including prolonged trial duration compared to a standard randomised controlled trial, time-varying confounding, instructing all parties involved, making sure each cluster is committed and performing the assigned policy, and communication with whole blood donors [14, 21]. Furthermore, the lack of blinding to the allocation for both participants and trial staff may introduce a risk of bias [22]. Recruitment bias for the questionnaire data is limited by inviting all eligible donors at the different measurement time points to participate. For other outcomes, all available information from donors who gave consent for their unusable remainder of the donation to be used for medical scientific research was used in the current study. Detection bias is expected to be minimal because outcome assessment does not include any judgement from the researchers [22]. For our sample size calculation, we considered multiple clustering effects that may be present in our study design. An individual autocorrelation coefficient will not affect our sample size as there are no repeated measurements from the same participants. As participants in the same cluster are more alike than participants in another cluster, we included an intraclass correlation coefficient of 0.10. Finally, it may be necessary to take into account a cluster autocorrelation coefficient. However, the cluster autocorrelation coefficient may be difficult to determine and the impact on the total sample size is probably limited [23]. Although the appropriate sample size calculation for stepped wedge designs is still subject of discussion, it is a potential limitation that we did not take into account a cluster autocorrelation coefficient in our sample size calculation. The setting of Sanquin as one organisation with multiple blood collection centres has some advantages in the execution of a stepped wedge cluster randomised trial compared to locally organised general practices or hospitals, for example. The blood collection centres are randomised to implementation steps and instructed to perform the assigned policy, with centralised organisation and monitoring. To our knowledge, this method has never been used in transfusion-related research. The effectiveness of the ferritin-guided donation interval policy on Hb and ferritin levels, Hb deferral rates, donor return rates and donor health likely has a delayed response, because (1) donors’ ferritin levels are only measured when they make a fifth donation and (2) the policy involves donation interval extension which delays follow-up. Delaying the implementation period of ferritin-guided donation intervals should therefore have minimal negative impact on the outcome of the study. Sanquin’s Ethical Advisory board approved the FIND’EM study protocol after the start of the implementation period and we were therefore not able to collect baseline questionnaire data in September 2017. Furthermore, this together with logistic constraints made it impossible to collect data at each implementation step. Therefore, we might have less detailed data on the effects of the new policy on the outcomes over time compared to a design with outcome measurements at each cross-over period. The secondary outcome donor health is measured at mid-implementation and after complete implementation. Investigating the effectiveness of the ferritin-guided donation intervals policy on donor health is limited to the use of questionnaire data collected at mid-implementation and after the implementation period. Data on ferritin levels is available at baseline, mid-implementation and after complete implementation of the policy.

Worldwide, increasing numbers of blood services consider implementing routine ferritin measurements in blood donors, though the effectiveness of ferritin-guided donation intervals has not yet been evaluated. Iron supplementation is an often-applied alternative intervention to limit iron deficiency in whole blood donors. Iron supplementation may increase iron stores in a shorter amount of time, thereby allowing blood donors to continue donating according to their regular donation interval [24]. Although some studies have evaluated the effect of oral iron supplementation on ferritin levels in blood donors, the recommended dosage and frequency of iron supplementation is unknown and the donors’ perception of iron supplementation should be further investigated. Overall, there is a general lack of evidence and consensus on the appropriate ferritin monitoring policy.

The FIND’EM study will provide ground-breaking evidence on the effectiveness of ferritin-guided donation intervals on Hb and ferritin levels, low-Hb deferral, donor return rates and donor health. This evidence will help blood services worldwide to improve donor iron management policies in order to protect and improve the health of donors.

## Trial status

At the time of manuscript submission, all clusters have implemented the ferritin-guided donation interval policy. Data collection at mid-implementation has been completed and data collection after completion of the implementation is planned for the end of November 2019. Ferritin measurements from stored blood samples are scheduled for 2020. The research team will communicate the results of the FIND’EM study via a final report without publication restrictions.

Protocol version 3, June 16, 2020

## Supplementary information

**Additional file 1.** Cognitive Failure Questionnaire (CFQ).

## Data Availability

The datasets that will be used and analysed during the current study will be available from the corresponding author on reasonable request.
