# Supplementary material for: Ferritin measurement IN Donors—Effectiveness of iron Monitoring to diminish iron deficiency and low haemoglobin in whole blood donors (FIND’EM): study protocol for a stepped wedge cluster randomised trial
Source: Trials. 2020 Oct 1;21:823. doi: 10.1186/s13063-020-04648-w (PMC7528293; doi:10.1186/s13063-020-04648-w)
Supplement: Supplementary file 1 — Additional file 1. Cognitive Failure Questionnaire (CFQ). [file 13063_2020_4648_MOESM1_ESM.pdf]

<u>Cognitive Failure Questionnaire (CFQ)</u>

Invulinstructie: *De volgende 25 vragen gaan over kleine, alledaagse vergissingen die iedereen van tijd tot tijd maakt. Sommige van die vergissingen overkomen u waarschijnlijk wat vaker dan andere. Hieronder kunt u het cijfer omcirkelen dat hoort bij uw antwoord. Hierbij is 1 voor 'zeer vaak', 2 voor 'vaak', 3 voor 'af en toe', 4 voor 'zelden' en 5 voor 'nooit'.*

1.	Iets lezen en vlak daarna niet meer weten wat u nu gelezen hebt, zodat u het moet overlezen	1	2	3	4	5
2.	Vergeten waarom u naar een bepaald gedeelte van uw huis bent gelopen	1	2	3	4	5
3.	Wegwijzers over het hoofd zien	1	2	3	4	5
4.	Links en rechts verwarren bij het beschrijven van een route	1	2	3	4	5
5.	Per ongeluk tegen mensen opbotsen	1	2	3	4	5
6.	Niet meer weten of u het licht of het gas hebt uitgedaan, of de deur hebt afgesloten	1	2	3	4	5
7.	Niet luisteren naar de naam van een persoon op het moment dat deze persoon zich aan u voorstelt	1	2	3	4	5
8.	Iets er uitflappen en achteraf bedenken dat dat wel eens beledigend voor iemand zou kunnen zijn	1	2	3	4	5
9.	Niet merken dat iemand iets tegen u zegt als u met iets anders bezig bent	1	2	3	4	5
10.	Boos worden en daar later spijt van hebben	1	2	3	4	5
11.	Belangrijke brieven dagenlang onbeantwoord laten	1	2	3	4	5
12.	Vergeten welke straat u moet inslaan als u een route kiest die u goed kent, maar die u maar zelden gebruikt	1	2	3	4	5
13.	In een supermarkt niet kunnen vinden wat u zoekt terwijl het er wel is	1	2	3	4	5
14.	U plotseling afvragen of u een woord op de juiste manier gebruikt	1	2	3	4	5
15.	Moeite hebben met het nemen van een beslissing	1	2	3	4	5
16.	Afspraken vergeten	1	2	3	4	5
17.	Vergeten waar u iets hebt neergelegd, zoals een boek of een krant	1	2	3	4	5
18.	Per ongeluk iets weggooien dat u nodig hebt en bewaren wat u weg wilde gooien	1	2	3	4	5
19.	Dagdromen terwijl u eigenlijk naar iets of iemand zou moeten luisteren	1	2	3	4	5
20.	Namen van mensen vergeten	1	2	3	4	5
21.	Beginnen met iets maar het niet afmaken, omdat u ongemerkt met iets anders bent begonnen	1	2	3	4	5
22.	Niet op een woord kunnen komen terwijl het 'op het puntje van uw tong' ligt	1	2	3	4	5
23.	In een winkel vergeten wat u kwam kopen	1	2	3	4	5
24.	Dingen uit uw handen laten vallen	1	2	3	4	5
25.	In een gesprek niets meer weten om over te praten	1	2	3	4	5

Extra vragen (NB: Als u bij alle voorgaande 25 vragen een 5 hebt omcirkeld, dan kunt u onderstaande vragen overslaan)

26. Is het maken van deze alledaagse vergissingen in de afgelopen 5 jaar bij u toegenomen?
    (1) helemaal niet toegenomen
    (2) een klein beetje toegenomen
    (3) matig toegenomen
    (4) nogal sterk toegenomen
    (5) zeer sterk toegenomen

27. Hoeveel hinder hebt u van het maken van deze vergissingen in het dagelijks leven?
    (1) helemaal geen hinder
    (2) zeer weinig hinder
    (3) een beetje hinder
    (4) veel hinder
    (5) zeer veel hinder

28. In hoeverre maakt u zich zorgen over het maken van deze vergissingen in het dagelijks leven?
    (1) helemaal geen zorgen
    (2) zeer weinig zorgen
    (3) een beetje zorgen
    (4) veel zorgen
    (5) zeer veel zorgen

29. Kunt u aangeven in hoeverre u zich ergert aan het maken van deze alledaagse vergissingen?
    (1) het ergert mij helemaal niet
    (2) het ergert mij een beetje
    (3) het ergert mij matig
    (4) het ergert mij nogal veel
    (5) het ergert mij zeer veel

Subschaal 'Verstrooidheid': vraag 2, 6, 16, 17, 18, 23, 24,
Subschaal 'Verstrooidheid in sociale situaties: vraag 8, 9, 10, 19
Subschaal 'Namen en Woorden': vraag 7, 20, 22
Subschaal 'Oriëntatie': vraag 3, 4, 12
